# Development and Validation of a Brief Version of the Research Engagement Survey Tool

**DOI:** 10.3390/ijerph181910020

**Published:** 2021-09-23

**Authors:** Melody S. Goodman, Nicole Ackermann, Kristyn A. Pierce, Deborah J. Bowen, Vetta Sanders Thompson

**Affiliations:** 1 School of Global Public Health, New York University, New York, NY 10003, USA; kp2224@nyu.edu; 2 Division of Public Health Sciences, School of Medicine, Washington University in St. Louis, St. Louis, MO 63110, USA; nackermann@wustl.edu; 3 Department of Bioethics and Humanities, University of Washington, Seattle, WA 98195, USA; dbowen@uw.edu; 4 Brown School, Washington University in St. Louis, St. Louis, MO 63130, USA; vthompson22@wustl.edu

**Keywords:** stakeholder engagement, survey scale, validation, research engagement

## Abstract

The Research Engagement Survey Tool (REST) examines the level of partner engagement in research studies. This study used mixed methods, including web-based surveys (N = 336), a modified Delphi process (N = 18), and cognitive response interviews (N = 16), with convenience sampling to develop and validate a short version of the REST. We conducted factor analysis and calculated internal consistency for the condensed REST. We validated the condensed REST against the comprehensive REST. All analyses were carried out on two scales (quality and quantity) based on Likert-type response options. We examined convergent validity with other measures theoretically associated with the REST (e.g., the Community Engagement Research Index and the Partnership Self-Assessment Tool). This study produced a 9-item condensed version of the REST. The condensed REST loads on 1 factor, has high internal consistency (Cronbach’s alpha = 0.92 for the quantity scale; 0.94 for the quality scale), is significantly correlated (ρ = 0.97; *p* < 0.001 for both scales) with the comprehensive (32-item) REST, and has negligible, low, and moderate correlation with other measures (e.g., the Partnership Assessment In community-based Research, trust in medical researchers, and the Coalition Self-Assessment Survey). Use of the condensed REST will reduce participant burden and time to complete. This standardized and validated quantitative measure is useful to compare engagement across projects or within a project over time.

## 1. Introduction

In our previous work, we developed and validated the comprehensive (32-item) Research Engagement Survey Tool (REST) to examine the level of non-academic stakeholder engagement among research partners [1,2,3,4]. The REST is based on eight engagement principles (EPs) from the research literature on community engagement, community–academic partnerships, community-based participatory research, and patient-centered outcomes research [1]. The tool was refined through a modified Delphi process to reach consensus among community and academic experts on each principle’s name and definition in addition to the 3 to 5 items used to assess each principle [1].

Standardized and validated survey tools are necessary to advance the science of stakeholder engagement to allow movement of the field beyond best practices and lessons learned to the development and implementation of evidence-based approaches for stakeholder engagement. The 32-item REST allows for comparisons of partner engagement across studies and within studies over time. The REST is measured on two Likert-type scales (quantity and quality), has high internal consistency, and is correlated with other theoretically related constructs. Research teams that have implemented the REST liked the comprehensiveness, the ease of administration and completion, and the usefulness as a starting point for conversations with their partners about engagement practices and goals.

However, the length of some survey scales can be burdensome, especially when combined with other survey items and scales that contribute to the overall time it takes a participant to complete a survey. Therefore, many researchers have developed short-form (SF) versions of commonly used questionnaires to reduce these burdens while still adequately measuring the constructs of interest.

Several different approaches have been used to develop shorter versions of commonly used survey scales in the public health and clinical literature. Arozullah and colleagues [5] developed and validated a brief version of the 66-item Rapid Estimate of Adult Literacy in Medicine (REALM). Using data from a sample of patients, the researchers first conducted a stepwise multiple regression analysis to determine the independent association between each REALM item (coded dichotomously, correct/incorrect) and the total REALM score. An additional stepwise multiple regression analysis was then conducted to select seven REALM-SF items with a priori item retention criteria of experiment wise *p* < 0.05 with each item explaining greater than 1% of the variation in total REALM scores. Several goodness-of-fit tests were conducted to confirm that the 1 factor solution adequately fit the set of seven dichotomous variables. Finally, Pearson correlations were computed between REALM and REALM-SF scores, and REALM-SF scores were mapped to mirror those from the original REALM instrument based on distributional similarities, which demonstrated excellent agreement [5].

Ware and colleagues [6] constructed an SF version of the comprehensive questionnaire used in the Medical Outcomes Study (MOS); the SF version is referred to as the SF-36. The researchers started by selecting the most frequently included health concepts in commonly used health surveys. To select individual items for inclusion in the SF-36 scales, they used the corresponding, comprehensive MOS scale as the criterion, with the goal of mirroring the original scale as closely as possible. Lastly, they correlated the scores of the new, shortened scales with the original scales to confirm agreement between the two scales [6].

Bobrovitz and colleagues [7] derived a short-form version of the 35-item self-completed, 28-item telephone interview, the Quality of Trauma Care Patient-Reported Experience Measure (QTAC-PREM). Using only complete cases of validation study data, the researchers conducted factor analyses in order to identify subscales of the instrument. Next, they used Cronbach’s alpha to examine the internal consistency of these previously identified subscales. Finally, to confirm the subscales were adequate predictors of the overall ratings of quality of care, univariate and multivariate associations were calculated utilizing Spearman’s correlations and ordinal regression. The resulting consolidated measure was shortened by 23 items, producing the QTAC-PREM short-form, with 24 self-completed items, followed by a 16-item telephone interview [7].

Cane and colleagues [8] developed and evaluated a short-form version of the Patterns of Activity Measure—Pain (POAM-P), which contains 30-items within three scales. Item-total correlations between each item and its corresponding scale were calculated. The items with moderate- to high-correlations with their corresponding scales were then assessed for their correlation to their two non-corresponding scales and retained as to reduce inter-scale correlation. Scale lengths were selected based on whether additional items affected a scale’s internal consistency or inter-relatedness of the scales. Items were retained to ensure key aspects of each scale were assessed. The resulting POAM-P-SF contained three scales, with 5 items in each scale (half the length of the original measure) [8].

Rush and colleagues [9] decided to consolidate the Quality of Life Enjoyment and Satisfaction Questionnaire Short-Form (Q-LES-Q-SF) using data from a sample of people who completed the Q-LES-Q-SF and the Quick Inventory of Depressive Symptoms-Self-Report (QIDS-SR) at two time points. Of the 16 items in the Q-LES-Q-SF, only the first 14 were considered for the Mini-Q-LES-Q. The seven items with the largest magnitude of change from beginning to end of this study, and the strongest correlation with the QIDS-SR, were included. To ensure goodness of fit, researchers compared the total sums of the items in the Mini-Q-LES-Q to those in the Q-LES-Q-SF, as well as the change in both measures with the change in the QIDS-SR [9].

SF versions of commonly used measures are beneficial for several reasons. First is their potential to decrease the amount of time required from a participant to complete the questionnaire, thus reducing burden on the participant. In busy settings, even a questionnaire that takes only 2 to 3 min can be prohibitive for interventional studies [5,7]. In addition, a shorter questionnaire can reduce cognitive burden for individuals already experiencing limited cognitive functioning due to some outside influence [8]. Aside from reducing burden for the participant, shorter measures are more acceptable to participants because they appear to be less redundant [6]. SF measures can be beneficial for researchers to efficiently obtain and compare data. With fewer metrics, results can be summarized more concisely, and reporting can be clearer and easier [7]. With these “snapshots” of the results, researchers can also quickly make decisions regarding how to respond (e.g., increase quality of life for the participants) if indicated by the measure [9].

Here, we discuss the development and validation of a short form of the REST using a mixed-methods triangulation analysis. A short form of the REST will allow project teams to assess engagement with fewer items and a shorter time to complete. Given the lack of a gold standard in the assessment of research partner engagement, triangulation analysis seems most appropriate for short form measure development, as it requires the use of team science approaches with multiple team members examining multiple pieces of data from a diverse group of stakeholders in order to make key decisions on item reduction.

## 2. Materials and Methods

We performed a mixed-methods study (using both quantitative and qualitative research approaches) that consisted of a modified Delphi process (conducted between July 2017 and August 2018) [1,2], cognitive interviews (conducted in October 2018) [4], and 4 longitudinal web-based surveys (conducted between July 2017 and September 2019). All participants were currently or previously involved in community-engaged research [1,2,3,4]. We used triangulation analysis on data from the Delphi process, cognitive interviews, and web surveys to guide the development of a brief version of the REST.

### 2.1. Longitudinal Web-Based Surveys

#### 2.1.1. Participants

Participants were recruited by several different methods throughout the study period, including virtual recruitment and in-person recruitment. Virtual recruitment included sending emails to those within the research team’s network and contacts identified in Clinical and Translational Science Awards Programs, Prevention Research Centers, Transdisciplinary Research in Energetics and Cancer Centers, National Institute on Minority Health and Health Disparities Centers of Excellence, National Cancer Institute Community Networks Programs, U.S. Department of Health & Human Services Regional Health Equity Councils, and health departments. In-person recruitment consisted of attending local health fairs and community meetings, posting flyers, and attending national conferences.

#### 2.1.2. Procedures

After participants were deemed eligible for this study, they were emailed a personalized link to the first web survey. Participants were emailed the links to the subsequent surveys either when the survey was launched or, if the participant enrolled in this study after the survey launch, after completion of the previous survey. If a participant did not complete the previous survey, they were still sent the subsequent survey so that all participants received an emailed invitation to all 4 surveys. Participants received $10 per survey for completing surveys 1 and 2, and an extra $5 if they completed both surveys 1 and 2; $15 per survey for completing surveys 3 and 4, and an extra $10 if they completed both surveys 3 and 4, for a total of $65 possible compensation for completing 4 surveys.

#### 2.1.3. Measures

The final comprehensive version of the REST that resulted from the modified Delphi process and cognitive interviews was administered to participants on the fourth longitudinal web survey. The comprehensive version of the REST is based on 8 EPs, with 3 to 5 items per engagement principle (EP), for a total of 32 items. The REST was presented to participants on 2 scales: quantity (“Please rate how often the partners leading the research do each of the following”) and quality (“Please rate how well the partners leading the research do each of the following”). The response options for the quantity scale were never, rarely, sometimes, often, always, and not applicable. The response options for the quality scale were poor, fair, good, very good, excellent, and not applicable. Responses were coded in order from 1 to 5 for both scales, with higher scores indicating higher engagement; not applicable options were coded as missing. For the REST, mean scores were calculated overall and by EP for both quality and quantity scales. The overall mean scores for both scales were created by averaging the mean EP scores so that each EP was weighted equally regardless of the number of items.

We compared the condensed REST to other similar measures captured throughout the longitudinal web surveys. On survey 1, we administered measures of medical mistrust [10], trust in medical researchers [11], a survey of community engagement from Kagan et al. [12], and the Partnership Assessment In community-based Research (PAIR) [13]. On survey 2, we administered the Community Engagement Research Index (CERI) [14] and the trust measure of the Coalition Self-Assessment Survey (CSAS) [15].

We administered the Partnership Self-Assessment Tool (PSAT) [16,17] and the Wilder Collaboration Factors Inventory [18,19] on survey 3. The PSAT includes measures of 11 dimensions—synergy, leadership, efficiency, administration and management, non-financial resources, financial resources, decision making, benefits, drawbacks, comparing benefits and drawbacks, and satisfaction. Additional details on these measures are provided in the Supplemental Materials in Table S1.

Demographic questions (i.e., age, gender, race, ethnicity, education level, and region) were collected on survey 1; however, if a participant had not previously responded to survey 1, the demographic questions and project description questions were asked of the participant on whichever survey they completed first. Age was measured continuously in years. Gender was coded as male, female, or other. Race and ethnicity were combined into categories of Non-Hispanic/Latino(a) Black, Non-Hispanic/Latino(a) White, Hispanic, Asian, and Other/Multiracial/Unknown. Education level was coded as less than high school, high school degree or GED, some college or associates degree, college degree, or graduate degree. Region was coded as Northeast, West, South, Midwest, and non-state area (includes Virgin Islands and Puerto Rico).

### 2.2. Modified Delphi Process

#### 2.2.1. Participants and Procedures

The modified Delphi process consisted of experts in stakeholder engagement in research who were academic researchers (N = 8) or community health stakeholders (N = 10). The 5-round Delphi panel process consisted of a series of web-based surveys and an in-person meeting with a goal of reaching agreement (80% or higher) among panelists on the EP names, definitions and items. The Delphi panel process has been described in detail elsewhere [1,2].

#### 2.2.2. Measures

We considered 2 metrics from the Delphi panel process when creating the condensed version of the REST. On surveys from rounds 2 and 3, we asked panelists to rank the EPs, with 1 being the most important and 8 being the least important. On the round 3 survey, we also asked panelists to select the 3 EPs with the greatest importance and the 3 EPs with the least importance.

### 2.3. Cognitive Interviews

#### 2.3.1. Participants and Procedures

We conducted cognitive interviews after the modified Delphi panel process but before the fourth longitudinal web-based survey. Participants (N = 16) who had previously been involved in community- or stakeholder-engaged research attended an in-person semi-structured interview that lasted 90 to 120 min. In an effort to identify potential modifications to be made to the measure, we aimed to use the cognitive interviews to determine how participants responded to and understood the items in the measure. The cognitive interview process has been described in detail elsewhere [3,4].

#### 2.3.2. Measures

We asked participants to rate item importance and difficulty answering the item. Participants were asked to rate the importance of each item for measuring community engagement by selecting 1 of the following response options: not at all important, slightly important, moderately important, very important, and extremely important. They were also asked to select a difficulty level from the following response options: extremely easy, somewhat easy, neither easy nor difficult, somewhat difficult, and extremely difficult.

### 2.4. Data Analysis

Triangulation refers to using more than one particular approach when doing research in order to get richer, fuller data and to help confirm the results of the research. Four different types of triangulation exist: data triangulation, investigator triangulation, theory triangulation, and methodological triangulation. Data triangulation (i.e., using different sources of data) includes different times for data collection, different places from which to collect the data, and different people who could be involved in the research study [20,21]. In investigator triangulation, several people are involved in the data gathering and data analysis processes (team science). In theory triangulation, multiple theories or perspectives are used in approaching the data to “extend the possibilities for producing knowledge” [21]. In methodological triangulation, several methods are used so that multiple perspectives are available on an issue being studied. Methodological triangulation can be applied either within method, by combining several perspectives, or between methods, by using several methods in a study [19,20,21,22]. We implemented triangulation (i.e., data, investigator, methodological) approaches using data from the modified Delphi process, longitudinal surveys, and cognitive interviews to develop the condensed REST.

Based on the results of the aforementioned mixed-methods analyses, items were dropped from the comprehensive version of the measure to create a condensed version of the measure. Criteria we investigated included a high amount (more than 5%) of not applicable or missing responses; cross-loading, non-loading, or low loadings in the factor analysis (using a cutoff value of 0.4); narrow distribution of responses (i.e., having a lower standard deviation as compared to other items); and lower slopes (value less than 1) or a more narrow range of threshold values from item response theory (IRT) models, as compared to other items in the measure. Additionally, we also took into consideration item importance and difficulty rankings from the cognitive interviews and item agreement as well as the ranking of EP importance from the modified Delphi process.

To compare the condensed version of the REST to the comprehensive version of the REST, we calculated Spearman’s correlation coefficients between measures and by individual EPs. We also calculated the mean difference between comprehensive and condensed versions, in addition to the mean square error, the mean absolute difference, and the mean relative absolute difference. We tested for significant differences in the medians between the comprehensive measure and condensed versions overall and for each EP using a Wilcoxon Signed Rank test. To compare the condensed version of the REST with other similar measures, we computed Spearman’s correlation coefficients. All analyses were conducted on both the quality and quantity scales of the REST. All statistical analyses were conducted in SAS**^®^** version 9.4. The institutional review boards at both Washington University in St. Louis and New York University approved all portions of this project.

## 3. Results

### 3.1. Longitudinal Web-Based Surveys

A total of 336 participants completed longitudinal web survey 4 (Table 1). Participants were mostly Non-Hispanic/Latino(a) Black (44%) or Non-Hispanic/Latino(a) White (42%), female (80%), had a graduate degree (45%), and were from the Midwest region of the United States (55%). The mean age of participants was 41 years (standard deviation: 14 years).

#### 3.1.1. Exploratory Factor Analysis

We conducted exploratory factor analysis on the comprehensive (32-item) REST. Items from the quality version of the REST loaded onto two factors, whereas items from the quantity version of the REST loaded onto four factors (Supplemental Table S2). For the quality scale of the REST, items 7.1 (All partners can use knowledge generated from the partnership), 2.1 (All partners have the opportunity to share ideas, input, and leadership responsibilities and to share in the determination of the project structure), 2.2 (Plans are developed and adjusted to meet the needs and concerns of the community or patient population), and 8.5 (All partners understand the culture of the organizations and community(ies) involved in the partnership) cross-loaded onto both factors. For the quantity scale, items 5.2 (The team works with existing community groups and organizations), and 5.3 (The team includes representation from the local community or patient population), 7.3 (All partners have the opportunity to be coauthors when the work is published) loaded onto more than 1 factor, and items 1.1 (The focus is on problems important to the community) and 7.1 (All partners can use knowledge generated from the partnership) did not load onto any factors. On the basis of analysis, these items were considered for exclusion from the condensed REST.

#### 3.1.2. Item Response Theory

Results from the IRT model showed slopes ranging from 0.92 (least informative) to 1.80 (most informative) for quantity items and slopes ranging from 1.17 (least informative) to 1.84 (most informative) for quality items. For the quality scale, the 5 items determined least informative (lowest slope value) were items 1.2, 1.3, 5.3, 7.3, and 8.4. For the quantity scale, the 5 items determined least informative (lowest slope value) were items 1.4, 5.3, 1.2, 1.3, and 5.2 (Supplemental Table S3). Based on this analysis, the items considered most informative were considered for inclusion in the condensed REST, and those considered least informative were considered for exclusion from the condensed REST.

### 3.2. Modified Delphi Process

Delphi panelists (*n* = 18) were majority female (94%) and African American or Black (61%), and all had some college or more education (100%). Delphi panelists had a range of research experience from 0 to 35 years (mean 14 years) and community-based participatory research experience from 0 to 30 years (mean 12 years).

#### Importance Ranking of Engagement Principle

The Delphi panel ranked EP1 (Focus on community perspectives and determinants of health) as the most important EP (rank 1) and EP7 (Involve all partners in the dissemination process) as the least important EP (rank 8) on both surveys in which they were asked to rank the EPs. In addition, on the round 3 survey where panelists were asked to list the 3 EPs of greatest importance and least importance, EP1 was in the 3 of greatest importance for 15 of the 18 panelists (83%)—this was the highest percentage for any EP. This was followed by EP2 (Partner input is vital) at 56% and EP8 (Build and maintain trust in the partnership; 44%). EP7 received the highest percentage of panelists listing as least important (83%), followed by EPs 3 (Partnership sustainability to meet goals and objectives) and 6 (Facilitate collaborative, equitable partnerships), both with 44% listing 1 of the EPs as 1 of the 3 least important EPs. Based on this analysis we deemed it important to include items from EPs 1, 2, and 8 in the condensed REST and items from EP7 were excluded from the condensed REST.

### 3.3. Cognitive Interviews

Cognitive interview participants (*n* = 16) were majority female (69%), African American or Black (69%), and had a college degree or higher level of education (56%).

#### 3.3.1. Item Importance Ranking

In the cognitive interviews, most respondents rated items as being very important or extremely important to community engagement (75–100%). Only three items received less than 75% of respondents rating them as very important or extremely important: items 4.1 (All partners have a variety of opportunities to gain new skills or knowledge from their involvement), 4.4 (All partners share resources to increase ability to address the problem of interest), and 7.3 (All partners have the opportunity to be coauthors when the work is published). Given their relative lack of importance, these items were considered for exclusion in the condensed REST.

#### 3.3.2. Item Difficulty Ratings

For the majority of items (*n* = 29, 91%), 25% or fewer respondents rated the items as somewhat to extremely difficult to answer. Only three items had over 25% of respondents rate the items as somewhat to extremely difficult: 1.2 (All partners look at the data to determine the health problems the community thinks are important), 4.3 (The partnership adds value to the work of all partners), and 7.2 (All interested partners are involved in activities related to sharing results). These three items were excluded from the condensed REST.

### 3.4. Triangulation Analysis

On the basis of the results described above, we created the condensed REST. Items that were non-loading or were cross-loading in the factor analysis (supplemental Table S2) for either quality or quantity versions (1.1, 2.1, 2.2, 5.2, 5.3, 7.1, 7.3, 8.5), items with more than 5% of ‘not applicable’ responses (1.3, 3.5, 6.1, 6.4, 7.3, 8.2; Supplemental Table S4), and items with an IRT model slope (Supplemental Table S3) less than 1 (1.2, 1.3, 1.4, 2.3, 5.2, 5.3) were considered for removal.

This left us with the following 16 items: 2.4, 3.1, 3.2, 3.3, 3.4, 4.1, 4.2, 4.3, 4.4, 5.1, 6.2, 6.3, 7.2, 8.1, 8.3, and 8.4. Using these criteria, all items from EP1 would have been cut. However, because the Delphi panel consistently rated EP1 as the most important EP, we felt it necessary to include an item to represent EP1; we included item 1.1. Additionally, we decided to drop EP7 items completely from the condensed measure for the following reasons: (a) the Delphi panel consistently rated EP7 as the least important EP; (b) results described above suggest excluding the majority of EP7 items; (c) 2 of the 3 items cross-loaded onto multiple factors (see supplemental Table S2), 1 item with a high percentage of ‘not applicable’ responses; (d) and items 7.2 and 7.3 were rated as being slightly more difficult and slightly less important relative to other items. For EPs 3 and 4, we looked at results from all analyses, comparing items within EPs and considering conceptual meaning and interpretation of the items. On this basis, items 3.4. and 4.3 were included in the condensed REST.

The Condensed REST included nine items:

- 1.1—The focus is on problems important to the community.
- 2.4—All partners assist in establishing roles and related responsibilities for the partnership.
- 3.4—Community-engaged activities are continued until the goals (as agreed upon by all partners) are achieved.
- 4.3—The partnership adds value to the work of all partners.
- 5.1—The team builds on strengths and resources within the community or patient population.
- 6.2—All partners’ ideas are treated with openness and respect.
- 6.3—All partners agree on the timeline for making shared decisions about the project.
- 8.1—The partnership’s processes support trust among all partners.
- 8.3—Mutual respect exists among all partners.

Similar to the comprehensive REST, the condensed REST is measured on two scales (quantity and quality) using Likert-type response options.

### 3.5. Validation of the Condensed REST

The condensed REST showed acceptable internal consistency (Cronbach’s alphas > 0.9 on both scales), similar means and medians to the comprehensive measure, and very high statistically significant correlations with the comprehensive REST (*ρ* = 0.97, *p* < 0.001 on both scales; Table 2). Table 3 shows results comparing the condensed REST with the comprehensive REST by EP for both the quality and quantity scales. 

Table 4 shows correlations among the condensed measure and several other similar measures for convergent validity. The condensed REST showed statistically significant correlations with all measures for both the quality and quantity scales. The condensed REST had negligible correlation with medical mistrust (0.1), trust in medical researchers (0.2), CERI (0.2), and the drawbacks dimension of the PSAT (quality = −0.2; quantity = −0.3; Table 4). For the quality scale, the condensed REST had low correlation with the following measures: PAIR (0.4), Kagan measure (0.5), the non-financial resources dimension of the PSAT (0.5), financial resources dimension of the PSAT (0.3), and the decision-making dimension of the PSAT (0.5; Table 4). The condensed REST had low correlation with the following measures for the quantity scale: PAIR (0.5), the Coalition Self-Assessment Survey—Trust subscale (0.4), benefits dimension of the PSAT (0.4), comparing benefits and drawbacks dimension of the PSAT (0.4), and the financial resources dimension of the PSAT (0.3).

The condensed REST had moderate correlation with the Wilder Collaboration measure (quality = 0.5; quantity = 0.6) and several dimensions of the PSAT (i.e., synergy [0.6], satisfaction [0.6], leadership [0.7], efficiency [0.6], administration/management [0.6]). For the quantity scale only, the condensed REST had moderate correlation with non-financial resources (0.5) and decision making (0.5) dimensions of the PSAT (Table 4). These convergent validity results are similar to results of comparisons between the various measures and the comprehensive version of the REST.

## 4. Discussion

Several approaches have been used to develop shorter versions of commonly used survey scales. Although no standard approach to item reduction exists, most of the literature shows a combination of statistical and theoretical approaches, including item-total correlations [8], internal consistency using Cronbach’s alpha [7,8], factor analysis [5,7], correlations (e.g., Spearman, Pearson) [5,6,7], regression [5,7], differences over time [9], assurance that the aspect of each scale is assessed [8], consideration of only some items [9], and inclusion of key concepts. The sample sizes vary across validation studies, some studies used only complete case data for validation [7], at a single time point [5,6,7,8], and others compared changes between time points [9].

We used data from a mixed-methods (surveys, cognitive interviews, modified Delphi process) study design and triangulation analysis to develop the short (9-item) version of the REST. The use of survey data analysis is similar to other approaches in the literature (e.g., internal consistency, item-total correlation, correlation between short and long forms), but the inclusion of transformed data (in quantitative formats) [23,24] from the cognitive interviews and modified Delphi process (qualitative research methods) strengthen this work.

### 4.1. Limitations

This work should be considered in the context of several limitations. First, all of our research approaches (surveys, Delphi process, cognitive interviews) used a convenience sampling recruitment methodology and is subject to the limitations of such samples in terms of generalizability. Second, recruitment delays for the longitudinal survey caused some participants to take the surveys out of order (*n* = 31; 6%). Third, a large proportion of those that screened eligible did not take any of the surveys (*n* = 94; 19%). Fourth, as with any longitudinal study, there was participant attrition. Among participants that completed at least one survey (*n* = 393), 85% completed the final longitudinal survey. Fifth, the addition of the “not applicable” response option increased the amount of missing data as those that reported not applicable were treated as missing in the data analysis. In addition, the REST is only available in English, and we did not estimate the time to complete.

Although there are several benefits to using an SF version of a measure, several limitations should also be considered. With fewer items, a measure will be more sensitive to change because of a single item [9]. When a scale is shortened, there is a trade-off between comprehensiveness of the measure and the measure’s precision in measuring each concept. In addition, shortened versions of measures may have floor and ceiling effects. This means that there is a possibility that a substantial percent of respondents will have the lowest possible and highest possible score, respectively [6].

### 4.2. Future Research

The REST was designed to be partnership, population, and health condition agnostic. However, future work should examine the fidelity of the REST to tailoring for specific populations, projects, partnerships, and health conditions. It may be important for future research to determine whether REST can predict the functioning of a multiple setting study, where there are a variety of community connections among the members. In addition, future work on the condensed REST should examine implementation of the measure in practice and examine the scoring and alignment with community engagement classification levels (e.g., coordination, cooperation, consultation, partnership) [3]. Future work should also ensure that the results of the REST implementation and analysis are accessible to lay audiences, specifically research partners. Future work should be carried out to adapt, validate, and establish the fidelity of the REST in languages other than English.

## 5. Conclusions

Given the trade-offs between comprehensive and short versions of survey scales, we believe that this condensed (9-item) version of the REST provides a nice complement to the comprehensive (32-item) version of the REST, and we recommend research teams use the version most appropriate for their project at each time point. The use of multiple forms of triangulation analysis to develop the condensed version of the REST allows stronger validity of the results despite the limitations of each approach (e.g., use of convenience samples, web access necessary for participation, transformation to quantitative data from qualitative research methods). The condensed REST is highly correlated with the comprehensive (32-item) version of the REST, has high internal consistency, and aligns on 1 factor.

## Figures and Tables

**Table 1:** Demographic Characteristics of Participants Who Completed Longitudinal Web Survey 4, Delphi Panelists, and Cognitive Interview Participants.

Category	Characteristic	Longitudinal Web Survey 4 (N = 336) n (%)	Delphi Panel (N = 18) n (%)	Cognitive Interviews (N = 16) n (%)
Race	Non-Hispanic/Latino(a) Black	147 (43.8)	11 (61.1)	10 (62.5)
	Non-Hispanic/Latino(a) White	140 (41.7)	6 (33.3)	4 (25.0)
	Hispanic/Latino(a)	17 (5.1)	1 (5.3)	1 (6.3)
	Asian	16 (4.8)	0 (0.0)	0 (0.0)
	Other/Multiracial/Unknown	16 (4.8)	0 (0.0)	1 (6.3)
Gender	Male	69 (20.5)	1 (5.6)	2 (12.5)
	Female	267 (79.5)	17 (94.4)	13 (81.3)
	Other/Unknown	0 (0.0)	0 (0.0)	1 (6.3)
Education	Less than HS	2 (0.6)	0 (0.0)	0 (0.0)
	HS degree or GED	12 (3.6)	0 (0.0)	2 (12.5)
	Some college or Associate degree	73 (21.7)	3 (16.7)	4 (25.0)
	College degree	97 (28.9)	1 (5.6)	2 (12.5)
	Graduate degree	152 (45.2)	14 (77.8)	7 (43.8)
	Unknown	0 (0.0)	0 (0.0)	1 (6.3)
Region	Midwest	183 (54.5)	8 (44.4)	--
	North East	39 (11.6)	2 (11.1)	--
	South	82 (24.4)	5 (27.8)	--
	West	31 (9.2)	3 (16.7)	--
	Virgin Islands	1 (0.3)	0	--
Age, median (range), y (n = 334)		41.1 (18–80)	51.9 (26–76)	47.3 (24–73)
		Mean (SD)		
Health literacy—SILS (possible range 1–5) ^a^				
Confident with Forms (n = 326)		4.4 (0.8)	--	--
Problems Reading (n = 326)		4.5 (0.9)	--	--
Help Read (n = 326)		4.4 (0.9)	--	--
Numeracy (n = 326) ^a^				
SNS ability subscale average (possible range 1–5)		3.9 (0.9)	--	--
SNS preference subscale average (possible range 1–6)		4.6 (1.0)	--	--

^a^ Higher scores indicate higher numeracy or health literacy. Note: SILS—Single Item Literacy Screener; SNS—Subjective Numeracy Scale GED—General Education Development Certificate (high school equivalent).

**Table 2:** Condensed REST and Comprehensive REST Comparison.

Variable	Number of Items	Cronbach’s Alpha	Mean	Standard Deviation	Median	Minimum	Maximum	Spearman’s Correlation with Comprehensive Version
Quantity Scale (n = 336)								
Condensed Version	9	0.92 ^a^	3.98	0.77	4.07	1.33	5.0	0.97 (*p* < 0.001)
Comprehensive Version	32	0.97 ^a^	3.92	0.74	4.04	1.51	5.0	---
Quality Scale (n = 332)								
Condensed Version	9	0.94 ^a^	3.72	0.92	3.86	1	5	0.97 (*p* < 0.001)
Comprehensive Version	32	0.98 ^a^	3.69	0.89	3.84	1	5	--

^a^ No items increase alpha if dropped.

**Table 3:** Condensed REST and Comprehensive REST Comparison by Engagement Principle.

Comparison	Condensed Mean (SD)	Comprehensive Mean (SD)	Mean Difference (SD)	Mean Square Error	Mean Absolute Difference	Mean Relative Absolute Difference	Spearman’s Correlation
Quantity							
Overall	4.0 (0.8)	4.0 (0.8)	0.04 (0.18)	0.03	0.13	0.04	0.97 (<0.001)
EP1: Focus on community perspectives and determinants of health	4.1 (0.9)	4.0 (0.8)	0.17 (0.56)	0.35	0.4	0.11	0.76 (<0.001)
EP2: Partner input is vital	3.8 (1.0)	3.9 (0.8)	−0.11 (0.53)	0.29	0.36	0.1	0.86 (<0.001)
EP3: Partnership sustainability to meet goals and objectives	3.8 (1.1)	3.7 (0.9)	0.04 (0.53)	0.28	0.37	0.11	0.87 (<0.001)
EP4: Foster co-learning, capacity building, and co-benefit for all partners	4.1 (0.9)	4.0 (0.8)	013 (0.46)	0.23	0.33	0.09	0.84 (<0.001)
EP5: Build on strengths and resources within the community or patient population	4.0 (1.0)	4.0 (0.9)	−0.03 (0.53)	0.28	0.32	0.09	0.86 (<0.001)
EP6: Facilitate collaborative, equitable partnerships	4.0 (0.9)	3.9 (0.9)	0.11 (0.31)	0.11	0.21	0.06	0.93 (<0.001)
EP8: Build and maintain trust in the partnership	4.0 (0.9)	4.0 (0.9)	0.03 (0.31)	0.1	0.22	0.06	0.93 (<0.001)
Quality							
Overall	3.7 (0.9)	3.7 (0.9)	0.03 (0.19)	0.04	0.14	0.04	0.97 (*p* < 0.001)
EP1: Focus on community perspectives and determinants of health	3.8 (1.1)	3.7 (0.9)	0.08 (0.56)	0.32	0.39	0.12	0.85 (<0.001)
EP2: Partner input is vital	3.6 (1.1)	3.7 (1.0)	−0.11 (0.54)	0.3	0.37	0.11	0.86 (<0.001)
EP3: Partnership sustainability to meet goals and objectives	3.6 (1.2)	3.6 (1.0)	0.04 (0.52)	0.27	0.35	0.11	0.90 (<0.001)
EP4: Foster co-learning, capacity building, and co-benefit for all partners	3.8 (1.1)	3.7 (1.0)	0.12 (0.48)	0.24	0.33	0.1	0.88 (<0.001)
EP5: Build on strengths and resources within the community or patient population	3.7 (1.1)	3.8 (1.0)	−0.06 (0.46)	0.22	0.28	0.08	0.89 (<0.001)
EP6: Facilitate collaborative, equitable partnerships	3.7 (1.0)	3.6 (1.0)	0.06 (0.33)	0.11	0.21	0.07	0.94 (<0.001)
EP8: Build and maintain trust in the partnership	3.8 (1.1)	3.8 (1.0)	0.01 (0.36)	0.13	0.23	0.07	0.93 (<0.001)

Note: EP—Engagement Principle.

**Table 4:** Condensed REST Convergent Validity with Other Measures.

Other Measures	Quality			Quantity		
	N	Spearman’s Ρ	*p* Value	N	Spearman’s Ρ	*p* Value
Medical Mistrust	322	0.11	0.04	325	0.14	0.01
Trust in Medical Researchers	322	0.17	0.002	324	0.21	<0.001
Partnership Assessment In community-based Research (PAIR)	322	0.41	<0.001	325	0.45	<0.001
Kagan Survey of community engagement	319	0.46	<0.001	322	0.52	<0.001
Community Engagement in Research Index (CERI)	320	0.17	0.003	323	0.21	<0.001
Coalition Self-Assessment Survey—Trust ^a^	323	0.38	<0.001	328	0.43	<0.001
Partnership Self-Assessment Tool (PSAT)						
PSAT—Synergy	325	0.60	<0.001	328	0.62	<0.001
PSAT—Satisfaction	324	0.59	<0.001	327	0.63	<0.001
PSAT—Leadership	323	0.68	<0.001	326	0.68	<0.001
PSAT—Efficiency	323	0.60	<0.001	326	0.57	<0.001
PSAT—Administration/Management	323	0.61	<0.001	326	0.63	<0.001
PSAT—Non-Financial Resources	322	0.46	<0.001	325	0.53	<0.001
PSAT—Financial and Other Capital Resources	320	0.33	<0.001	323	0.33	<0.001
PSAT—Decision Making	325	0.49	<0.001	328	0.51	<0.001
PSAT—Benefits	324	0.32	<0.001	327	0.40	<0.001
PSAT—Drawbacks	324	−0.20	<0.001	327	−0.27	<0.001
PSAT—Comparing Benefits and Drawbacks	323	0.40	<0.001	326	0.44	<0.001
Wilder Collaboration	325	0.53	<0.001	328	0.55	<0.001

^a^ Correlation with EP8 (Build and maintain trust in the partnership) only. Note: Spearman’s Ρ is a correlation coefficient. It is the nonparametric counterpart of the Pearson’s correlation coefficient.

## Data Availability

Data are available in ICPSR: (https://www.openicpsr.org/openicpsr/workspace?goToPath=/openicpsr/126361&goToLevel=project; accessed on 4 August 2021).

## Supplementary Materials

The following are available online at www.mdpi.com/article/10.3390/ijerph181910020/s1, Table S1: Comparison Measures, Table S2: Factor Analysis Results—Comprehensive Version of REST, Table S3: Item Response Theory Results—Comprehensive Version of REST title, Table S4: Comprehensive REST Item Information Summary.
