# Supplementary material for: Development and Validation of a Brief Version of the Research Engagement Survey Tool
Source: Int J Environ Res Public Health. 2021 Sep 23;18(19):10020. doi: 10.3390/ijerph181910020 (PMC8507896; doi:10.3390/ijerph181910020)
Supplement: Supplementary file 1 [file ijerph-18-10020-s001.zip › ijerph-1348211-supplementary.pdf]

Table S1. Comparison Measures

Name	Domain	Longitudinal survey	Number of items	Scoring/ interpretation	Validation (*if applicable*)
Mainous Trust in Medical Researchers [10]	Trust in medical researchers	1	12	Score ranging from 0 to 48; higher values indicate higher trust	Cronbach's alpha = 0.84
Hall Trust in Medical Researchers [11]	Trust in medical researchers	1	12	Score ranging from 0 to 100; higher values indicate higher trust	Cronbach's alpha = 0.87. Factor model consists of 1 factor.
Survey of community engagement [12]	Community engagement	1	3 categories; 25 items	Average score ranging from 1 to 7; higher scores indicate higher engagement	n/a
Partnership Assessment In community-based Research (PAIR) [13]	Evaluates key dimensions of researchers and community member partnerships	1	5 dimensions; 31 items	Average score ranging from 1 to 5; higher scores indicate higher engagement	Content validation (literature review, experts, cognitive interviews)
Community Engagement Research Index (CERI) [14]	Community engagement in research	2	12	Sum score ranging from 4 to 12; higher scores indicate	Face validity (items identified by interview participants), content

Table S1. Comparison Measures

Name	Domain	Longitudinal survey	Number of items	Scoring/ interpretation	Validation (*if applicable*)
				higher engagement	validity (items based on previously collected qualitative data)
Coalition Self-Assessment Survey (CSAS) trust sub-scale	Trust within coalition	2	7	Average score ranging from 1 to 4; higher scores indicate higher trust	No validation data, but used across several projects
The Community Campus Partnerships for Health (CCPH) principles	Community campus partnerships	2	10 principles; 47 items		No validation data, but used across several projects
Partnership Self-Assessment Tool (PSAT) – Synergy	Partnership synergy	3	9	Average score ranging from 1 to 5, rounded to 0.1; higher scores indicate higher synergy	According to the National Collaborating Centre for Methods and Tools (NCCMT), the PSAT tool has been evaluated, validity properties meet accepted standards,
Partnership Self-Assessment Tool (PSAT) – Leadership	Partnership leadership	3	11	Average score ranging from 1 to 5, rounded to 0.1; higher scores	

Table S1. Comparison Measures

Name	Domain	Longitudinal survey	Number of items	Scoring/ interpretation	Validation (*if applicable*)
				indicate better leadership	and reliability properties meet accepted standards. They gave the tool a 'strong' methodological rating.
Partnership Self-Assessment Tool (PSAT) – Efficiency	Partnership Efficiency	3	3	Average score ranging from 1 to 5, rounded to 0.1; higher scores indicate better efficiency	
Partnership Self-Assessment Tool (PSAT) – Administration/ Management	Partnership administration/ management	3	9	Average score ranging from 1 to 5, rounded to 0.1; higher scores indicate better administration/ management	
Partnership Self-Assessment Tool (PSAT) – nonfinancial resources	Partnership nonfinancial resources	3	6	Average score ranging from 1 to 5, rounded to 0.1; higher scores indicate has resources	
Partnership Self-Assessment Tool (PSAT)	Partnership financial other	3	3	Average score ranging from 1 to 5, rounded to	

Table S1. Comparison Measures

Name	Domain	Longitudinal survey	Number of items	Scoring/ interpretation	Validation (*if applicable*)
– financial other capital resources	capital resources			0.1; higher scores indicate has resources	
Partnership Self-Assessment Tool (PSAT) – decision making	Partnership decision making	3	3	Average score ranging from 1 to 5, rounded to 0.1; higher scores indicate better decision making	
Partnership Self-Assessment Tool (PSAT) – benefits	Partnership benefits	3	11	Percentage score (out of nonmissing items); higher score indicates more benefits	
Partnership self-assessment tool (PSAT) – Drawbacks	Partnership drawbacks	3	6	Percentage score (out of nonmissing items); higher score indicates more drawbacks	
Partnership Self-Assessment Tool (PSAT) – comparing	Comparing benefits and drawbacks	3	1	1='Drawbacks greatly exceed the benefits' to 5='Benefits greatly exceed the drawbacks'	

Table S1. Comparison Measures

**Name**	**Domain**	**Longitudinal survey**	**Number of items**	**Scoring/ interpretation**	**Validation (*if applicable*)**
benefits and drawbacks					
Partnership Self-Assessment Tool (PSAT) – satisfaction	Partnership satisfaction	3	5	Average score ranging from 1 to 5, rounded to 0.1; higher scores indicate higher satisfaction	
Wilder Collaboration Factors Inventory	Collaboration evaluation	3	6 dimensions, 20 factors, 40 items	Average score ranging from 1 to 5 with dimensions equally weighted; higher scores indicate higher collaboration	Reliability varied for dimensions (alpha 0.50 – 0.93) [19]

Table S2. Factor Analysis Results – Comprehensive Version of REST

Item Number	Item	Quantity (n=234)				Quality (n=224)	
		**F1**	**F2**	**F3**	**F4**	**F1**	**F2**
1.1	The focus is on problems important to the community.						*X*
1.2	All partners look at the data to determine the health problems the community thinks are important.	*X*				*X*	
1.3	The effort incorporates factors (for example housing, transportation, food access, education, employment) that influence health status.				*X*		*X*
1.4	The focus is on cultural factors that influence health behaviors.				*X*		*X*
2.1	All partners have the opportunity to share ideas, input, and leadership responsibilities and to share in the determination of the project structure.	*X*				*X*	*X*
2.2	Plans are developed and adjusted to meet the needs and concerns of the community or patient population.				*X*	*X*	*X*
2.3	All partners agree to take on specific tasks according to their comfort, ability, and expertise.	*X*				*X*	
2.4	All partners assist in establishing roles and related responsibilities for the partnership.	*X*				*X*	
3.1	All partners share updates, progress, strategies, and new ideas regularly.	*X*				*X*	
3.2	A plan is in place for ongoing problem-solving.	*X*				*X*	
3.3	All partners are involved in determining next steps.	*X*				*X*	
3.4	Community-engaged activities are continued until the goals (as agreed upon by all partners) are achieved.	*X*				*X*	

Table S2. Factor Analysis Results – Comprehensive Version of REST

Item Number	Item	Quantity (n=234)				Quality (n=224)	
		F1	F2	F3	F4	F1	F2
3.5	All partners continue community-engaged activities beyond an initial project, activity, or study.	*X*				*X*	
4.1	All partners have a variety of opportunities to gain new skills or knowledge from their involvement.			*X*			*X*
4.2	All partners are encouraged to learn from each other.			*X*			*X*
4.3	The partnership adds value to the work of all partners.			*X*			*X*
4.4	All partners share resources to increase ability to address the problem of interest.			*X*			*X*
5.1	The team builds on strengths and resources within the community or patient population.			*X*			*X*
5.2	The team works with existing community groups and organizations.			*X*	*X*		*X*
5.3	The team includes representation from the local community or patient population.			*X*	*X*		*X*
6.1	Fair processes have been established to manage conflict or disagreements.	*X*				*X*	
6.2	All partners ideas are treated with openness and respect.		*X*			*X*	
6.3	All partners agree on the timeline for making shared decisions about the project.	*X*				*X*	
6.4	All partners agree on ownership of data for publications and presentations.	*X*				*X*	
7.1	All partners can use knowledge generated from the partnership.					*X*	*X*
7.2	All interested partners are involved in activities related to sharing results.	*X*				*X*	

Table S2. Factor Analysis Results – Comprehensive Version of REST

Item Number	Item	Quantity (n=234)				Quality (n=224)	
		**F1**	**F2**	**F3**	**F4**	**F1**	**F2**
7.3	All partners have the opportunity to be coauthors when the work is published.	*X*	*X*			*X*	
8.1	The partnerships processes support trust among all partners.		*X*			*X*	
8.2	All partners are confident that they will receive credit for their contributions to the partnership.		*X*			*X*	
8.3	Mutual respect exists among all partners.		*X*			*X*	
8.4	All partners respect the population being served.		*X*				*X*
8.5	All partners understand the culture of the organizations and community(ies) involved in the partnership.		*X*			*X*	*X*
Eigenvalue		20.2	1.45	1.18	1.03	22.9	1.08
Variance Accounted for		0.63	0.05	0.04	0.03	0.72	0.03

Table S3. Item Response Theory Results – Comprehensive Version of REST

Item Number[a]	Quality			Quantity		
	IRT slope	Slope rank	IRT threshold range	IRT slope	Slope rank	IRT threshold range
1.1	1.34	23	3.24	1.05	25	3.77
1.2	1.28	28	2.84	0.95	30	3.53
1.3	1.23	31	3.13	0.96	29	3.66
1.4	1.30	27	2.88	0.92	32	4.12
2.1	1.72	4	2.48	1.34	17	3.50
2.2	1.69	5	2.83	1.61	2	3.11
2.3	1.51	18	3.00	0.98	27	3.20
2.4	1.56	14	3.04	1.43	7	2.93
3.1	1.54	16	2.99	1.37	15	2.91
3.2	1.60	9	2.63	1.36	16	2.99
3.3	1.64	6	2.58	1.53	5	3.32
3.4	1.84	1	2.52	1.58	3	2.65
3.5	1.47	19	2.45	1.09	24	3.05
4.1	1.64	7	2.77	1.21	21	3.49
4.2	1.57	12	2.71	1.29	19	3.09
4.3	1.60	8	2.92	1.41	10	3.10
4.4	1.47	21	2.82	1.38	13	2.96
5.1	1.72	3	2.61	1.54	4	2.51
5.2	1.33	25	2.69	0.98	28	3.16
5.3	1.24	30	3.26	0.92	31	3.63
6.1	1.34	24	2.92	1.24	20	3.01
6.2	1.54	17	2.79	1.42	9	3.49
6.3	1.58	11	3.06	1.40	11	3.66
6.4	1.47	20	2.87	1.31	18	2.82
7.1	1.45	22	2.65	1.44	6	2.71
7.2	1.60	10	2.38	1.43	8	2.49
7.3	1.17	32	2.46	1.01	26	3.03
8.1	1.84	2	2.70	1.80	1	2.91
8.2	1.55	15	2.33	1.37	14	2.50
8.3	1.56	13	3.01	1.39	12	2.76
8.4	1.24	29	2.95	1.20	22	2.74
8.5	1.31	26	2.82	1.18	23	3.13

Abbreviation: IRT, item response theory.

[a]See Table S2 for wording of full item.

Table S4. Comprehensive REST Item Information Summary

Item Number	Item text	Quantity					Quality				
		Mean	Std dev	Median	Not applicable		Mean	Std dev	Median	Not applicable	
					N	Percent				N	Percent
1.1	The focus is on problems important to the community.	4.10	0.85	4.0	3.0	0.9	3.73	1.03	4.0	5.0	1.5
1.2	All partners look at the data to determine the health problems the community thinks are important.	3.80	1.01	4.0	13.0	3.9	3.61	1.13	4.0	13.0	3.9
1.3	The effort incorporates factors (for example housing, transportation, food access, education, employment) that influence health status.	3.90	0.96	4.0	18.0	5.4	3.62	1.11	4.0	24.0	7.1
1.4	The focus is on cultural factors that influence health behaviors.	3.90	0.96	4.0	15.0	4.5	3.66	1.10	4.0	13.0	3.9
2.1	All partners have the opportunity to share ideas, input, and leadership responsibilities and to share in the determination of the project structure.	3.88	0.98	4.0	12.0	3.6	3.61	1.11	4.0	13.0	3.9
2.2	Plans are developed and adjusted to meet the needs and concerns of the	3.95	0.96	4.0	12.0	3.6	3.70	1.09	4.0	12.0	3.6

Table S4. Comprehensive REST Item Information Summary

Item Number	Item text	Quantity					Quality				
		Mean	Std dev	Median	Not applicable		Mean	Std dev	Median	Not applicable	
					N	Percent				N	Percent
	community or patient population.										
2.3	All partners agree to take on specific tasks according to their comfort, ability, and expertise.	3.90	0.96	4.0	10.0	3.0	3.61	1.07	4.0	11.0	3.3
2.4	All partners assist in establishing roles and related responsibilities for the partnership.	3.81	0.96	4.0	8.0	2.4	3.51	1.06	4.0	9.0	2.7
3.1	All partners share updates, progress, strategies, and new ideas regularly.	3.82	1.05	4.0	7.0	2.1	3.57	1.09	4.0	13.0	3.9
3.2	A plan is in place for ongoing problem-solving.	3.72	1.06	4.0	13.0	3.9	3.49	1.16	4.0	13.0	3.9
3.3	All partners are involved in determining next steps.	3.65	1.02	4.0	10.0	3.0	3.41	1.18	3.0	11.0	3.3
3.4	Community-engaged activities are continued until the goals (as agreed upon by all partners) are achieved.	3.75	1.09	4.0	10.0	3.0	3.53	1.16	4.0	10.0	3.0
3.5	All partners continue community-engaged activities beyond an initial project, activity, or study.	3.59	1.11	4.0	22.0	6.5	3.42	1.21	4.0	20.0	6.0
4.1	All partners have a variety of opportunities to gain	3.80	0.95	4.0	4.0	1.2	3.56	1.09	4.0	8.0	2.4

Table S4. Comprehensive REST Item Information Summary

Item Number	Item text	Quantity					Quality				
		Mean	Std dev	Median	Not applicable		Mean	Std dev	Median	Not applicable	
					N	Percent				N	Percent
	new skills or knowledge from their involvement.										
4.2	All partners are encouraged to learn from each other.	4.02	0.99	4.0	4.0	1.2	3.68	1.11	4.0	6.0	1.8
4.3	The partnership adds value to the work of all partners.	4.02	0.96	4.0	4.0	1.2	3.76	1.08	4.0	5.0	1.5
4.4	All partners share resources to increase ability to address the problem of interest.	3.80	1.01	4.0	7.0	2.1	3.65	1.09	4.0	12.0	3.6
5.1	The team builds on strengths and resources within the community or patient population.	3.88	1.05	4.0	9.0	2.7	3.61	1.11	4.0	11.0	3.3
5.2	The team works with existing community groups and organizations.	4.08	0.98	4.0	6.0	1.8	3.76	1.13	4.0	8.0	2.4
5.3	The team includes representation from the local community or patient population.	3.91	0.99	4.0	10.0	3.0	3.68	1.11	4.0	16.0	4.8
6.1	Fair processes have been established to manage conflict or disagreements.	3.71	1.06	4.0	21.0	6.3	3.48	1.14	4.0	24.0	7.1

Table S4. Comprehensive REST Item Information Summary

Item Number	Item text	Quantity					Quality				
		Mean	Std dev	Median	Not applicable		Mean	Std dev	Median	Not applicable	
					N	Percent				N	Percent
6.2	All partners' ideas are treated with openness and respect.	4.08	0.91	4.0	3.0	0.9	3.69	1.09	4.0	7.0	2.1
6.3	All partners agree on the timeline for making shared decisions about the project.	3.83	0.92	4.0	10.0	3.0	3.54	1.07	4.0	13.0	3.9
6.4	All partners agree on ownership of data for publications and presentations.	3.78	1.07	4.0	25.0	7.4	3.51	1.14	4.0	22.0	6.5
7.1	All partners can use knowledge generated from the partnership.	4.07	0.97	4.0	7.0	2.1	3.75	1.11	4.0	8.0	2.4
7.2	All interested partners are involved in activities related to sharing results.	3.76	1.09	4.0	9.0	2.7	3.63	1.17	4.0	10.0	3.0
7.3	All partners have the opportunity to be coauthors when the work is published.	3.47	1.19	4.0	36.0	10.7	3.20	1.32	3.0	41.0	12.2
8.1	The partnership's processes support trust among all partners.	3.89	0.95	4.0	9.0	2.7	3.65	1.11	4.0	9.0	2.7
8.2	All partners are confident that they will receive credit for their contributions to the partnership.	3.71	1.13	4.0	18.0	5.4	3.46	1.25	4.0	19.0	5.7

Table S4. Comprehensive REST Item Information Summary

Item Number	Item text	Quantity					Quality				
		Mean	Std dev	Median	Not applicable		Mean	Std dev	Median	Not applicable	
					N	Percent				N	Percent
8.3	Mutual respect exists among all partners.	3.96	0.99	4.0	6.0	1.8	3.75	1.07	4.0	7.0	2.1
8.4	All partners respect the population being served.	4.12	0.98	4.0	8.0	2.4	3.90	1.08	4.0	8.0	2.4
8.5	All partners understand the culture of the organizations and community(ies) involved in the partnership.	3.78	1.02	4.0	7.0	2.1	3.67	1.12	4.0	9.0	2.7
